# Influence of Stress Level and Fibre Volume Fraction on Fatigue Performance of Glass Fibre-Reinforced Polyester Composites

**DOI:** 10.3390/polym14132662

**Published:** 2022-06-29

**Authors:** Mahmoud Yousry Zaghloul, Moustafa Mahmoud Yousry Zaghloul, Mai Mahmoud Yousry Zaghloul

**Affiliations:** 1 Department of Accounting, Faculty of Commerce, Alexandria University, Alexandria 21554, Egypt; business_workk@yahoo.com; 2 Department of Mechanical Engineering, Faculty of Engineering, Alexandria University, Alexandria 21554, Egypt; 3 Department of Chemistry and Physics, Faculty of Science, Alexandria University, Alexandria 21554, Egypt; chemistmai@yahoo.com

**Keywords:** fatigue, stress level, polyester, mechanical properties, GFRP, polymer composites, glass fibre

## Abstract

Fibre-reinforced polymeric composite materials are becoming substantial and convenient materials in the repair and replacement of traditional metallic materials due to their high stiffness. The composites undergo different types of fatigue loads during their service life. The drive to enhance the design methodologies and predictive models of fibre-reinforced polymeric composite materials subjected to fatigue stresses is reliant on more precise and reliable techniques for assessing their fatigue life. The influences of fibre volume fraction and stress level on the fatigue performance of glass fibre-reinforced polyester (GFRP) composite materials have been studied in the tension–tension fatigue scenario. The fibre volume fractions for this investigation were set to: 20%, 35%, and 50%. The tensile testing of specimens was performed using a universal testing machine and the Young’s modulus was validated with four different prediction models. In order to identify the modes of failure as well as the fatigue life of composites, polyester-based GFRP specimens were evaluated at five stress levels which were 75%, 65%, 50%, 40%, and 25% of the maximum tensile stress until either a fracture occurred or five million fatigue cycles was reached. The experimental results showed that glass fibre-reinforced polyester samples had a pure tension failure at high applied stress levels, while at low stress levels the failure mode was governed by stress levels. Finally, the experimental results of GFRP composite samples with different volume fractions were utilized for model validation and comparison, which showed that the proposed framework yields acceptable correlations of predicted fatigue lives in tension–tension fatigue regimes with experimental ones.

## 1. Introduction

Polymer matrix composite materials are being increasingly used for a variety of engineering and other scientific applications. They comprise of a fibre reinforcement phase ingrained into a matrix phase that is either a thermosetting polymer or a thermoplastic polymer. The diameter of the fibres can range from a nanometre to a few millimetres [1]. The benefits of fibre-reinforced composite materials include high elastic modulus and high specific strength, high resistance to fatigue and corrosion, high flexibility, high design flexibility, adequate resistance to wear and creep, facile fabrication, and being environmental friendly as well as economically efficient [2,3,4]. These characteristics make fibre-reinforced composite materials suitable for a diverse and wide array of applications in different sectors, such as high-performance engineering applications, military, energy, automotive industry, aerospace, construction and building, communication and networking, dentistry and orthopedics, and several other sectors [5,6,7,8,9,10].

Solid particles including glass or minerals are generally blended to reinforce Polymeric materials. These fillers can provide advantages to the procedure of manufacturing such as reduction in cost, enhanced processing, thermal and optical characteristics enhancements, density control, fire retardancy, thermal expansion control, enhancements in electrical and magnetic properties, and improved mechanical characteristics including hardness and resistance to fatigue [11,12,13,14]. Polymer matrix composite materials are generally used for either short fibre or continuous long fibre reinforcement. PMC materials, unlike other material types, are usually manufactured by the same process that achieves the final product; that is, the technique for producing the specific part also makes the composite material. Polyester materials are durable thermoset polymers that can be natural or synthetic and can be categorized as aliphatic or aromatic, depending on their backbone design [15]. Various studies have indicated the use of polyester materials in advanced engineering or high technology areas including shielding composites for electromagnetic interference, energy conversion devices, textile materials, and biomedical devices [16,17,18,19]. Subsequently, enhancements in polyester material including mechanical strength, heat stability, rheology, chemical stability, as well as glass transition temperature were made to increase its efficiency as a matrix that can be utilized in various areas [20,21].

The processing techniques for components subjected to light stresses were performed on a small scale, which included considerable parts of manual intervention in the processing. Low density, stiffness, and high strength of the raw materials was relied upon in order to reach to the required characteristics. As the complexity of geometries and the stress values increases, the manual involvement decreases noticeably, replaced by complex, sophisticated robotic techniques that account for delivering less defective, highly consistent, and enhanced manufacturing rates. Nevertheless, the concepts of these processes have been kept unmodified, and there are existing troubles in minimizing the production costs of these techniques [22,23,24]. These structures are usually subjected to fluctuating stresses that deteriorates the material, leading to its degradation because of fibre/matrix de-bonding, matrix cracking, and fibre fracturing [25].

The response of composite materials for an extended time of loading is critical since the FRP design for civil infrastructure is generally influenced by serviceability instead of strength. Hence, a deeper knowledge of the influence of fluctuating loads on the structural behavior of composite materials is required in order to verify their safety for the aimed design life [26]. The performance of GFRP demonstrates linearity and failure that is usually brittle, which makes them primarily distinctive from metals where material failure originates from a crack and propagation leads to failure. Subsequently, GFRP failure is abrupt with no warning. Therefore, comprehending its fatigue life corresponding to its design factors becomes essential. The characteristics of the fibre orientations, type of matrix, applied stress, frequency, and stress concentration are the main factors that can affect GFRP fatigue life [27]. The tension–tension fatigue performance of epoxy reinforced with flax fibres in diagonal, longitudinal, and transverse directions has demonstrated a loss in fatigue modulus of 10% to 55%, depending on the applied stress level and fibre orientations [28]. The research on fatigue damage growth performance of polymeric composite materials reinforced with carbon fibres suggested that the origination and propagation of fatigue cracks are highly conditional to the applied level of stress, and it was reported that transverse crack propagation and delamination were the main failure mechanisms [29]. Under fatigue loading, the average mechanical stress that distinguishes between tension–compression, tension–tension, or compression–compression fatigue loading regimes affects the mechanical characteristics of polymeric composites [30,31,32]. In addition, the damage mechanisms are affected by the applied stress level, the angle between the applied load, and the reinforcing fibres and the load amplitude, which have been thoroughly studied in other research studies. It was observed in previous research activities that damage mechanisms of polymers reinforced with unidirectional fibres highly depend on the fibre volume fraction in the tension–tension fatigue scenario, in addition to the previously mentioned factors [33,34,35]. The monitored damage mechanisms differ in specimens with high and low fibre volume fraction. While interfacial de-bonding and matrix cracking were the primary damage mechanisms in samples composed of low fibre volume fractions, interfacial de-bonding followed by fibre pull-out was observed in samples with a high fibre volume fraction, which leads to higher endurance limit.

The fatigue life prediction of composite structures is a notoriously tough activity, particularly when the structure consists of stress raisers or stress concentration activators such as holes, weld points, or cut-outs. Currently, there are very limited research outcomes that can be used with high reliability of concepts for predicting the fatigue performance of polymeric composite members. The design concepts utilized for polymeric composites are predominantly dependent on analytical models and experimental data [36,37,38,39], which are empirical essentially in their nature and usually only usable to certain materials, certain loading conditions, and specific stacking sequences of laminates. The lack in the prediction models of the fatigue life have led to design conservatism of composite members such as setting high safety factors. The present models for predicting fatigue life and material degradation due to fatigue stresses tended to be restricted to certain stacking sequences and loading conditions such as shear, compression, and tension. The models are limited to constant cyclic loading regimes and a certain frequency [40,41,42]. It is virtually impossible to extend the models to actual composite structures that are usually complex in geometry and assembly. The stress conditions imposed upon composite structures are additionally far more complicated due to them involving fluctuating applied forces with time. There are a few sets of spectral loading conditions that are characteristic to particular structures, such as FALSTAFF (Fighter aircraft loading Standard), WISPERX (Wind spectrum reference), and TWIST (Transport wing standard), which were designed to simulate the loading sequence for military aircraft and aircraft transport [43,44].

Several methods were applied to predict the fatigue life of the materials. A technique was deployed to monitor the temperature of the material surface [45,46,47,48]. The methods were based on the testing of the cooling properties of samples when they reached a steady temperature and actuation halted. It was shown that the cooling profile is unique for a particular geometry and material type irrespective of operating parameters such as the loading scenario, frequency, and amplitude. Modelling strategies for predicting the high-cycle fatigue performance of materials accurately based on the microstructural details, tensile characteristics, and loading parameters have been investigated [49,50,51,52,53,54,55,56,57]. The results demonstrated that the fatigue life of materials under a high-cycle fatigue regime can be accurately replicated and predicted through crack growth life. The overall model comprises of macroscopic finite element analysis, microstructural analysis, and crack growth analysis. The predicted results were in a very close agreement with the experimental results. Additionally, self-heating techniques, which are based on the measuring of temperatures, have been proposed as a quicker and more cost-efficient solution rather than conducting traditional fatigue testing [58]. One-dimensional thermo-mechanical constitutive formulas of shape memory alloys were implemented into the self-heating process by utilizing a two-scale probabilistic model for studying the fatigue performance of the materials. The model proved to be capable of predicting the fatigue strength and the number of cycles till failure for the materials in addition to the scattering of data by using probability equations.

In addition, non-destructive imaging methods showed a unique concept to investigate the crack initiation and crack propagation behaviours in structural materials [59]. Bragg edge transmission imaging was utilized to generate two-dimensional maps of the mean elastic strain, with sufficient spatial resolutions to visualize the effect and severity of fatigue cracks. Neutron computed tomography allows visualising the crack profiles as well as presenting information on the presence of several phases, and potentially on granular displacements. Nevertheless, extensive work is still needed to evaluate the fatigue damage progressing and development under complicated variable amplitude loading scenarios in order to enhance the reliability of the fatigue models for predicting the lifetime of polymeric composite materials. In order to broaden the comprehension of the damage of polymeric composite materials, this research studies the influence of fibre volume fractions and the applied stress level on the damage in glass fibre-reinforced polyester composites tested with fibre volume fractions of 20%, 35%, and 50%. The experimental tensile behaviour and tension–tension fatigue behaviour are assessed and different theoretical models are applied to predict Young’s modulus and the fatigue life of glass fibre-reinforced polyester composites. Finally, this research aims to predict and optimize the fatigue life of reinforced polymeric composites to be strong candidate materials in several industrial fields.

## 2. Materials and Methods

### 2.1. Materials

In this section, the procedures used to manufacture the required materials and the physical experiments conducted are outlined. It is vital to note that all mentioned experiments in this research were carried out at room temperature. The materials used in this investigation were classified into two main constituents: an unsaturated polyester resin as a matrix, and continuous E-glass fibres with dTex of 2400 as a reinforcement. The hardener and accelerator materials for the reaction were Methyl ethyl ketone peroxide and cobalt naphthenate, respectively. The mechanical and physical characteristics of the matrix and reinforcement materials were provided by the material suppliers and are presented in Table 1 and Table 2, respectively.

### 2.2. Manufacturing

The pultrusion process was used as the manufacturing method of the composite specimens as it is a cost-saving method for manufacturing steady, continuous cross-section composite material shapes [60,61]. Raw materials comprise of two phases: a matrix phase that is polyester, and a reinforcement phase that is represented by the E-glass fibres, which were varied with respect to the fibre volume fraction to 20%, 35%, and 50%. The continuous fibres were pulled off creels, where they passed into a bath that contained the polyester resin. The impregnated E-glass fibres were pulled in a mould that had the geometry of the final part, then, through a heated die, the composite parts were left to cure. As the E-glass fibres were impregnated with polyester, the polyester matrix was hardened as a result of the temperature generated in the forming die, and a rigid, cured shape was created that resembled the die geometry. Finally, the parts were post-cured in the oven at 50 °C for 6 h. For quality control and to verify the fibre volume fraction, a resin burn-off test was conducted according to ASTM D3171-99 on all produced parts. After conducting the resin burn-off test on all produced parts by physically removing polyester by ignition while leaving the glass fibres essentially unaffected, the amount of glass fibres were calculated experimentally by measuring the mass of fibres after ignition and mass of overall composite parts before ignition. The volumetric percentage of fibres was calculated for the three composite configurations according to Equation (1):

V_r_ = M_f_/M_i_ × 100 × ρ_c_/ρ_r_ (1)

where V_r_ is the volumetric percentage of glass fibres; M_f_ is the mass of glass fibres in grams; M_i_ is the initial mass of the specimen in grams; ρ_c_ is the density of the specimen in g/mL; and ρ_r_ is the density of glass fibres in g/mL.

**Figure 1:**
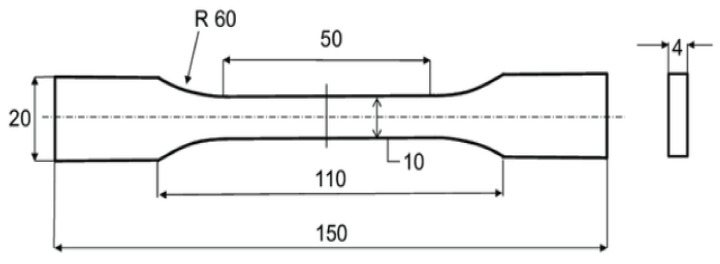
Specimen geometry in mm for tensile specimens according to ASTM D3039/D3039M.

**Figure 2:**
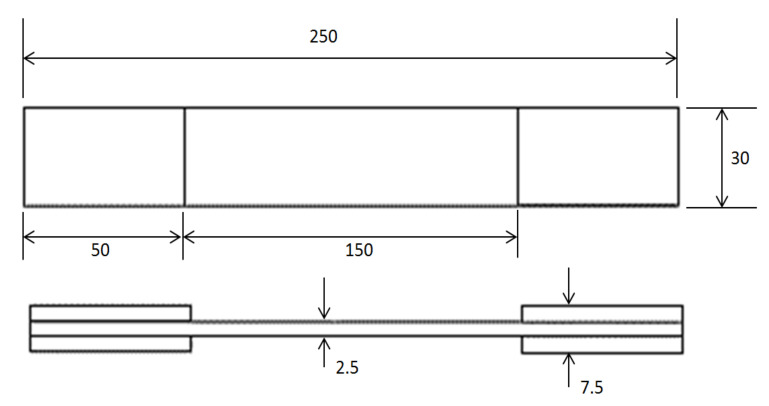
Specimen geometry for tension–tension fatigue specimens in mm according to ASTM D3479/D3479M.

The manufactured plates were cut using a water jet cutter to match the dimensions presented in Figure 1 and Figure 2 for obtaining the tensile and fatigue specimens.

### 2.3. Tensile Testing

The static tensile testing of the polyester-reinforced composite specimens was performed by using an Autograph AGS-X universal testing machine, whose capacity is 100 KN, supplied by Shimadzu (Tokyo, Japan), and the tensile testing of samples was examined at room temperature. The machine was calibrated before carrying out the experiments to ensure that the obtained results would be reliable. The standard dimensions of the tensile testing specimens for the polyester-reinforced composite specimens is presented in Figure 1, which are in accordance with the ASTM D3039/D3039M standard [62]. The samples were carefully clamped onto the machine jaws, and the tightening force applied on all specimens was constant in order to adhere to the bias and precision requirements of the ASTM standard for the testing of materials. The recording of data was applied by means of a video extensometer in order to overcome the slippage between the samples and grippers. The set value for the speed of the tensile test was 5 mm/min, as per the ASTM standard for tensile testing of polymeric composites. The Young’s modulus was evaluated from the linear portion of the stress–strain curve by dividing the stress over its corresponding strain at any given point on the linear region. The number of specimens used for each composition was five and the mean values of stiffness and ultimate tensile strength were recorded.

### 2.4. Tension–Tension Fatigue Testing

The geometry and dimensions of the tension–tension fatigue testing specimens for the polyester-reinforced composite specimens are presented in Figure 2, which are in accordance with the ASTM D3479/D3479M standard [63]. The tension–tension fatigue testing of polymer composite samples was carried out at five different stress levels which were 75%, 65%, 50%, 40%, and 25% of the maximum tensile load. The tension–tension fatigue testing was undertaken by utilizing an Instron servo-hydraulic universal testing machine (Instron, Norwood, MA, USA) with the load imposed through hydraulic grips with wedge-action. The tension–tension fatigue testing of polymer composite samples was performed under room temperature conditions, which was 25 ± 2 °C and 50 ± 10% relative humidity. The utilized stress ratio that is defined as the ratio between the minimum and maximum applied stresses was set to R = +0.15 for all testing conditions. The load and displacement for all compositions were recorded during the tension–tension fatigue testing. The test completion was decided when failure occurred or after reaching five million cycles, whichever came first. For statistical purposes and for the bias and precision standards, three identical specimens were examined at each of the five introduced stress levels in the low cycle fatigue regime test. The fracture surfaces of specimens after the tension–tension fatigue test were prepared for morphological analysis. The samples were coated with a platinum layer using a nano coater device, where the thickness of the applied coating layer was 10 nm and the coated samples were investigated by using a scanning electron microscope, SEM (JEOL JSM-5300, Tokyo, Japan), at an applied voltage of 8 kV.

## 3. Results

The average volumetric percentages (V_r_) of PE20GF, PE35GF, and PE50GF were calculated according to Equation (1) and the average V_r_ was found to be: 19.92%, 34.83%, and 49.87%, respectively.

In this section, the tensile behaviour of polyester composites as well as predictive models of Young’s modulus has been presented and analysed. In addition, the tension–tension fatigue of polyester composites and a predictive model of fatigue life have been explained.

### 3.1. Tensile Behaviour of Polyester Composites

**Figure 3:**
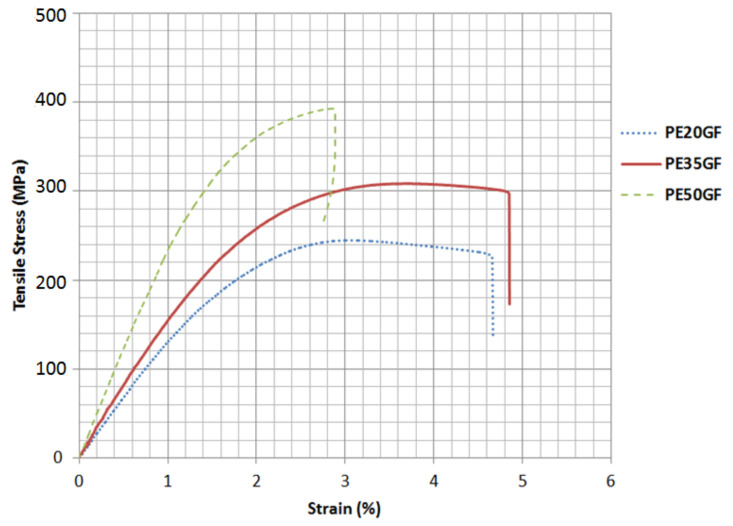
Stress–strain curve for polyester specimens reinforced with 20%, 35%, and 50% fibre volume fractions.

The tensile characteristics of the samples were assessed prior to conducting the fatigue testing in order to identify the corresponding load to any given stress level under fluctuating loads. The tensile behaviour of the polyester composite samples at the three fibre volume fractions is presented in Figure 3, in which each curve represents the average data points of five samples. The variance of the results was negligible; therefore, the average curve was drawn for the three different compositions. It can be observed that the increase in the fibre volume fraction led to a direct increase in the stiffness and ultimate tensile strength of the materials. The average and standard deviation (σ) in the ultimate tensile strength of PE20GF, PE35GF, and PE50GF were 242.8 MPa, σ = 21.73, 304.82 MPa, σ = 31.46, and 396.43 MPa, σ = 36.27, respectively. The ultimate tensile strength of PE50GF samples was higher than that of PE35GF and PE20GF by 27.4% and 63.8%, respectively. The composite specimens failed at a mean tensile load of 48.1 KN, 37.8 KN, and 29.6 KN for PE50GF, PE35GF, and PE20GF, respectively.

### 3.2. Relationship between Young’s Modulus and Fibre Volume Fraction

**Figure 4:**
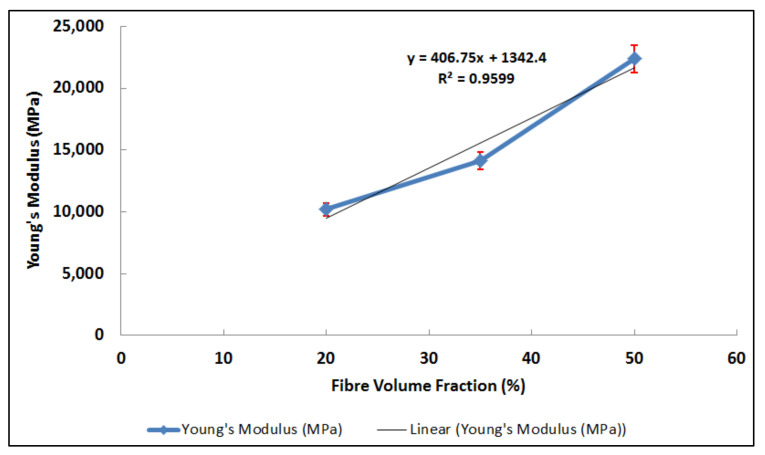
Young’s Modulus for polyester specimens reinforced with 20%, 35%, and 50% fibre volume fractions.

Young’s modulus values against the fibre volume fraction are represented in Figure 4. It can be seen that higher concentrations of fibre in the polyester matrix led to a significant increase in the Young’s modulus of the composite materials. The average and standard deviation (σ) in Young’s modulus of PE20GF, PE35GF, and PE50GF were 10.19 GPa, σ = 54.71, 14.13 GPa, σ = 76.01, and 187.37 GPa, σ = 54.71, respectively. The elastic modulus of PE50GF was higher than that of PE20GF and PE35GF by 52.9% and 81.4%, respectively. This increase in tensile modulus is attributed to the fact that the Young’s modulus of glass fibres is much higher than that of polyester matrix. The adhesion strength between the matrix and the fibres also contributes to this effect. The trend of modulus increase follows Equation (2) with R^2^ goodness of fit value equals to 0.9589, which shows that the model is closely fitting to the experimental data.

y = 406.75x + 1342.4 (2)

### 3.3. Relationship between the Ultimate Tensile Strength and Fibre Volume Fraction

**Figure 5:**
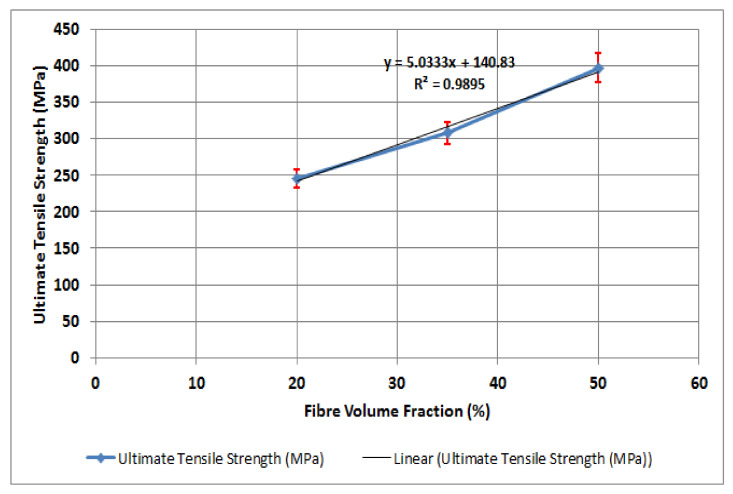
Ultimate tensile strength for polyester specimens reinforced with 20%, 35%, and 50% fibre volume fractions.

The ultimate tensile strength values against the fibre volume fractions are represented in Figure 5. It can be seen that higher concentrations of fibre in the polyester matrix led to a significant increase in the tensile strength of the composite materials. The ultimate tensile strength of PE50GF was higher than that of PE20GF and PE35GF by 28.6% and 62.5%, respectively. This increase in tensile modulus is attributed to the fact that the load capacity of glass fibres is much higher than that of polyester matrix. The adhesion strength between the matrix and the fibres also contributes to this effect. The trend of the ultimate tensile strength increase follows Equation (3) with R^2^ goodness of fit value equals to 0.9895, which shows that the model is only 1% deviated from the experimental data.

y = 5.0333x + 140.83(3)

### 3.4. Prediction Models for Young’s Modulus

In this section, three different models will be proposed for the prediction of the elastic modulus of polyester reinforced with glass fibres. The three models are the rule of mixtures, inverse rule of mixtures, and Halpin Tsai. The models are to be compared with the experimental values of Young’s modulus for the purpose of identifying the closely agreeing model to the actual values, which can be used as a prediction model for identifying the properties of relatively similar materials that are subjected to the same service conditions.

#### 3.4.1. Rule of Mixtures Model

The rule of mixtures is one of the primary series theoretical models used to predict the Young’s modulus of two-component composite materials, which usually over-predicts the actual figures. Equation (4) shows the Young’s modulus of composite materials as a function of fibres and matrix volume fractions and modulus.

(4) $$E_{c}=E_{f}V_{f}+E_{m}V_{m}$$

where,

E_c_ is the Young’s modulus of the composite materials.
E_f_ is the Young’s modulus of the fibres.
E_m_ is the Young’s modulus of the matrix.
V_f_ is the fibre volume fraction.
V_m_ is the matrix volume fraction, which is equal to 1 − V_f_.

#### 3.4.2. Halpin–Tsai Model

A versatile prediction model for assessing the tensile modulus of polymeric composite materials is Halpin–Tsai model [64]. The model reflects the properties of constituent materials, weight/volume fraction of the constituent materials, filler packing, and the aspect ratio of fillers. It provides a reliable estimation for the tensile modulus of nano-filled composite materials and is usually utilized to compare theoretical results with experimental results. There are two applied models, represented in Equations (5) and (6), which are the 2D and the 3D models for random orientations of fibres in two-dimensional space and random orientations of fibres in three-dimensional spaces, respectively.

(5) $$\eta =\frac{\beta \frac{E_{f}}{E_{m}}-1}{\beta \frac{E_{f}}{E_{m}}+2\alpha }$$

(6) $$E_{c}=\frac{1+{\alpha \eta V}_{f}}{1+{\eta V}_{f}}E_{m}$$

where, E_c_, E_m,_ and E_f_ are the composite Young’s modulus of the composite, Young’s modulus of the matrix, and Young’s modulus of the reinforcement, respectively, and the aspect ratio α, which is the ratio between length and diameter of reinforcing particles. In the 2D Halpin–Tsai model, β equals one, while in the 3D Halpin–Tsai model, β equals 1/6.

#### 3.4.3. Inverse Rule of Mixtures Model

The prediction of Young’s modulus figures of composite materials, consisting of a reinforcement phase and a matrix phase, is represented by the inverse rule of mixture as shown in Equation (7).

(7) $$\frac{1}{E_{c}}=\frac{V_{m}}{E_{m}}+\frac{V_{f}}{E_{f}}$$

where,

E_c_ is the Young’s modulus of the composite materials.
E_f_ is the Young’s modulus of the fibres.
E_m_ is the Young’s modulus of the matrix.
V_f_ is the fibre volume fraction.
V_m_ is the matrix volume fraction, which is equal to 1-V_f_.

### 3.5. Modelling of Young’s Modulus

The Young’s modulus of neat polyester and reinforced polyester with glass fibres at 25%, 35%, and 50% by fibre volume fraction were 0.91 GPa, 10.2 GPa, 14.1 GPa, and 22.4 GPa, respectively. Table 3 shows the measured values for the Young’s modulus of neat polyester and polyester-reinforced composites. Four prediction models for the modulus of elasticity were applied and it was found that the rule of mixtures model over-predicts the Young’s modulus values. On the other hand, the 3D Halpin–Tsai model and the inverse rule of mixtures model under predict the Young’s modulus values for the materials in this study. It was concluded that the 2D Halpin–Tsai model is the closest prediction model to the experimental data for representing the Young’s modulus of glass fibre-reinforced polyester composites.

### 3.6. Tension–tension Fatigue Behaviour of Polyester Composites

The samples at 75% and 65% of the maximum load were fractured when the fibres at the gauge length showed tensile failure and excessive sets of broken fibres and bundles. Nevertheless, when the samples were exposed to 50% and 40% of the maximum load, the failure was observed without scattered cuts in fibres. Moreover, the samples exposed to 25% of the maximum load did not encounter failure for up to 5 million fatigue cycles, and hence the test was stopped. Thus, it is clear that the stress concentration was fatigue failure mode; this type of failure occurs with high probability in case there is a rapid change in the geometry of the specimen or a change in material composition as well as subjecting the samples to high cycle fatigue conditions. It was observed that the slope of the load-displacement relationship gradually decreased, which indicated that the stiffness of the composite materials was being deteriorated. The glass fibre-reinforced polyester composite materials exhibited 91% retention of its initial strength after 5 million fatigue cycles for samples reinforced with 50% of glass fibres. The level of applied stresses, loss of stiffness in composite materials, the mean fatigue life, and the failure modes are tabulated in Table 4, which has the average values of three identical samples tested at each of the presented conditions.

**Figure 6:**
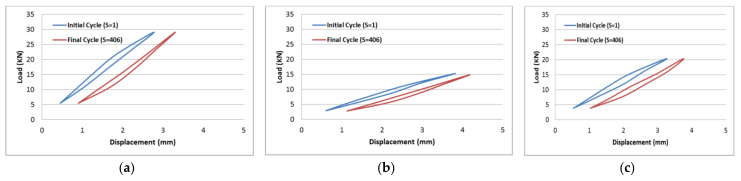
Load-displacement performance at 75% of the maximum load for fibre volume fraction (**a**) 50% glass fibre-reinforced polyester, (**b**) 35% glass fibre-reinforced polyester, and (**c**) 20% glass fibre-reinforced polyester.

**Figure 7:**
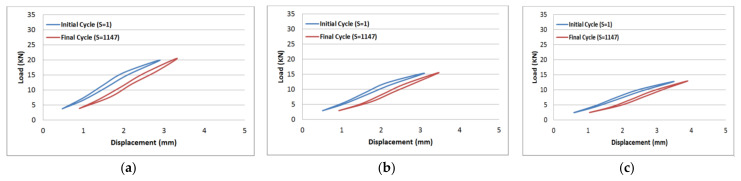
Load-displacement performance at 65% of the maximum load for fibre volume fraction (**a**) 50% glass fibre-reinforced polyester, (**b**) 35% glass fibre-reinforced polyester, and (**c**) 20% glass fibre-reinforced polyester.

**Figure 8:**
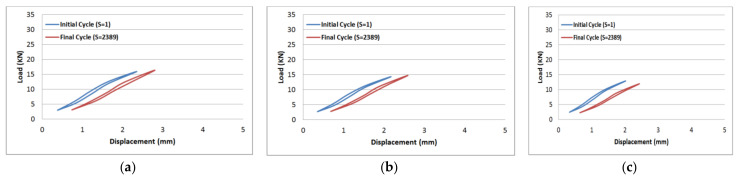
Load-displacement performance at 50% of the maximum load for fibre volume fraction (**a**) 50% glass fibre-reinforced polyester, (**b**) 35% glass fibre-reinforced polyester, and (**c**) 20% glass fibre-reinforced polyester.

**Figure 9:**
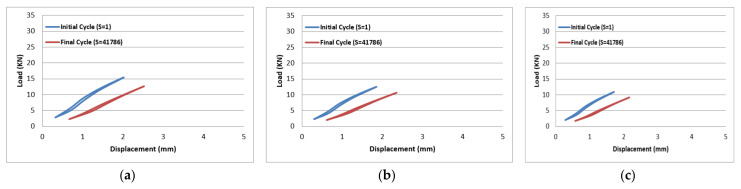
Load-displacement performance at 40% of the maximum load for fibre volume fraction (**a**) 50% glass fibre-reinforced polyester, (**b**) 35% glass fibre-reinforced polyester, and (**c**) 20% glass fibre-reinforced polyester.

**Figure 10:**
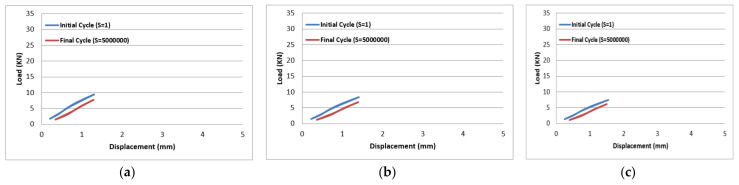
Load-displacement performance at 25% of the maximum load for fibre volume fraction (**a**) 50% glass fibre-reinforced polyester, (**b**) 35% glass fibre-reinforced polyester, and (**c**) 20% glass fibre-reinforced polyester.

The results of tension–tension fatigue testing on glass fibre-reinforced polyester samples at three fibre volume fractions are presented in five diagrams (Figure 6, Figure 7, Figure 8, Figure 9 and Figure 10). The previously mentioned figures represent the applied stress levels; 75%, 65%, 50%, 40%, and 25%, respectively. It can be observed that the fibre volume fraction influenced the load-displacement slope and the fatigue strength. The increase in the volume fraction of glass fibres from 20% to 50% led to an increase in the fatigue strength by 100.4% in the high stress level fatigue scenario. However, in the low stress level fatigue scenario, the increase in the volume fraction of glass fibres from 20% to 50% led to an increase in the fatigue strength by 38.2%.

**Figure 11:**
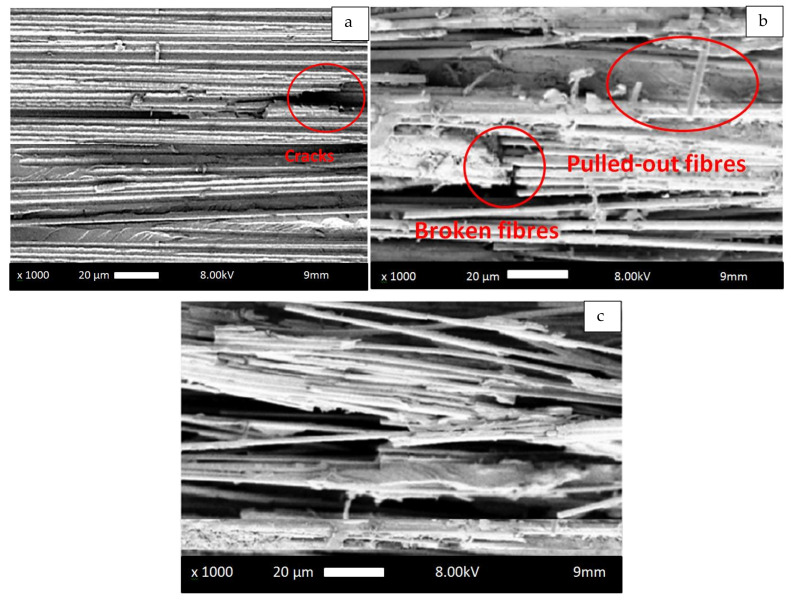
SEM micrographs of the fracture surfaces after tension–tension fatigue testing for (**a**) 20% glass fibre-reinforced polyester, (**b**) 35% glass fibre-reinforced polyester, and (**c**) 50% glass fibre-reinforced polyester.

The SEM micrographs presented in Figure 11 showed fibre pull-out as a primary damaging mechanism, which was accounted for in the transition of the specimens to higher strains. The fibre volume fraction primarily influenced the tension–tension fatigue testing in the high cycle fatigue scenarios because of the damage mechanisms transforming at high stress levels from fibre pull-out to matrix cracking at low stress levels.

The different damage mechanisms when comparing the samples with a low fibre volume fraction, represented in Figure 11a,b, to the sample with a high fibre fraction, represented in Figure 11c, prior to being subjected to tension–tension fatigue experiments are remarkable. The fibre volume fraction had an influence on the fracture surfaces where fibre bundles were broken and fragments of broken fibres were visible on the fracture surface. During tension, the load imposed on the specimens pulled the glass fibres out of the fracture plane, as observed in fatigue test with a stress ratio R = 0.15. The consequential tension load appeared to press the glass fibres, which resulted in either damaging the polyester matrix or due to pressing the fibres against each other. In this scenario, for samples containing a high fibre volume fraction, it is more probable that glass fibres were pressed against each other, which can even lead to localized bending load and accordingly lead to breakage of fibre bundles accompanied by fibre pull-out.

The found results and the attributed damage mechanisms of the three volumetric fractions of glass fibre-reinforced polyester composites, measured at five stress levels, are schematically represented in Table 5. The primary damage mechanisms in samples reinforced with low fibre volume fractions were fibre–matrix de-bonding and matrix cracking. In addition, the primary damage mechanism in samples reinforced with high fibre volume fractions was fibre pull-out in a direction perpendicular to the fracture plane, which resulted in increased endurance limit. The fibre volume fraction primarily affected the tension–tension fatigue testing of the polyester composite samples in the high cycle fatigue scenarios because of the change in the damage mechanisms from fibre pull-out at higher stress levels to matrix cracking at low stress levels, which is in close agreement with the results reported by Ansari et al. [65]. Moreover, the broken fibres were still bonded in the polyester matrix in compositions poor in fibre content, which was not the case with the pulled-out fibres for compositions rich in fibres. Additionally, the applied stress levels were found to magnify but not change the damage mode.

### 3.7. Modelling of the Fatigue Behaviour of Polyester Composites

Manson and Hertzberg [66] formulated the influence of stress ratio (R), maximum applied stress (σ_max_), and ultimate tensile strength (σ_u_) on the fatigue performance of GFR polyester composite materials subjected to fully reversed and tension–tension loading scenarios, as shown in Equation (8).

F (R, σ_u_, σ_max_) = σ_u_^1 − ϒ^ σ_max_^ϒ^ (1 − ψ)^ϒ^ (8)

The previous authors experimentally identified the quantity of the constant ϒ and found it to lie between 0.6 < ϒ < 7.6 for the propagation of damage in polymeric composite materials. Nevertheless, it can be evaluated from the smallest angle ϴ between the loading direction and the fibre direction, which is presented in Equation (9).

ϒ = 1.6 − ψ sin ϴ (9)

In Equations (8) and (9), ψ is defined as follows:

Ψ = R for −∞ < R < 1 [Reverse loading and tension–tension fatigue].

Ψ = 1/R for 1 < R < ∞ [Compression-compression fatigue].

The fatigue failure will happen when the maximum applied stress (σ_max_) approaches the ultimate tensile strength (σ_u_). The cycles count needed to lead to degradation in the strength of material from the ultimate tensile strength to the maximum applied stress is called the fatigue life, which can be represented by Equation (10).

(10) $$(\sigma )\;=\;\frac{C_{2}}{-m_{2}+1}\;(t^{-m_{2}+1})$$

where $-m_{2}$ and C_2_ are the material constants while t is the failure time.

With the re-arrangement of the fatigue life formula in Equation (10), the two parametric variables α and β can be introduced in Equations (11) and (12) as follows:

(11) $$\beta \;=\;-m_{2}+1$$

(12) $$\alpha =\frac{A}{-m_{2}+1}$$

Therefore, Equation (13) was obtained.

(13) $$(\frac{\sigma_{u}}{\sigma_{\max}}-1)\;(\frac{\sigma_{u}}{\sigma_{\max}}{)}^{ϒ-1}\frac{1}{{\left(1-\;R\right)}^{ϒ}}=(N^{\beta \;}\;-1)f^{-\;\beta }$$

where N is the number of cycles and f is the applied frequency

After further arrangement of Equation (13) to obtain the fatigue life (N) in the left hand side, Equation (14) was obtained.

(14) $$N=({\left(1+\frac{f^{\beta }}{\alpha }\;\left(\frac{\sigma_{u}}{\sigma_{\max}}-1\right)\left(\frac{\sigma_{u}}{\sigma_{\max}}{)}^{ϒ-1}\;\frac{1}{{\left(1-R\right)}^{ϒ}}\right)\right)}^{\frac{1}{\beta }}$$

Three fatigue testing values at two stress levels (75% and 40%) were used to identify the factors α and β. Equation (5) represents a linear relation showing a line that passes by the origin point when drawing the left hand side of the equation against the value ($N^{\beta }$ − 1)$f^{-\beta }$. The line with the best fit can be obtained after several runs to obtain the value of the parameters, which was β = 0.22475, while the gradient of the straight line that passes by the origin point was found to be α = 0.14331.

**Figure 12:**
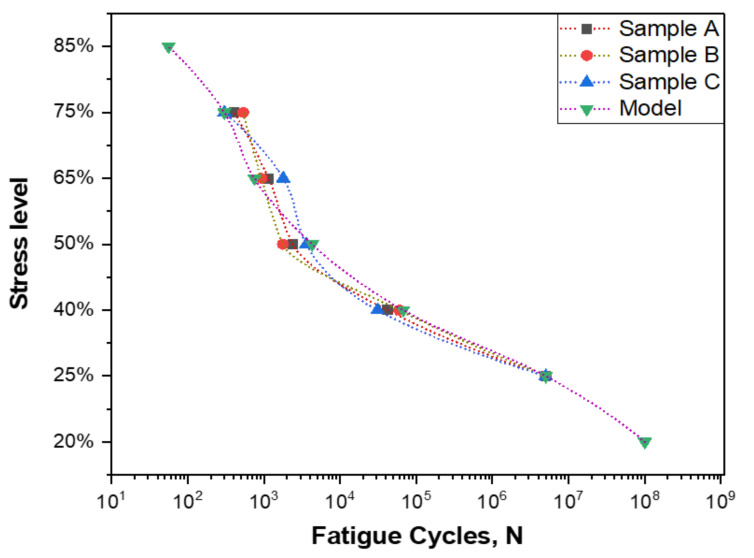
Influence of stress level on fatigue life of glass fibre-reinforced polyester composites; experimentally and theoretically.

The influence of the applied stress levels on the fatigue performance of GFR polyester composites is plotted in Figure 12, where three identical samples were tested for each stress level. The applied analytical model was found to be in close agreement with the experimental fatigue behaviour and was used to predict the fatigue life at very high stress levels such as at 85% of the ultimate load, as well as at very low stress levels such as at 20% of the ultimate load. It can be observed from the figure that the decrease in the stress level applied leads to a significant increase in the fatigue life of polyester composite materials; however, the outcome is slightly nonlinear even in semi-log fitting. The prediction accuracy of the presented model as calculated from the mean absolute percentage error for all data points at the different applied stress levels was found to be 89.42%.

Table 6 shows the intercept values for the fatigue life data of each sample as well as for the analytical model. In addition, the standard error, t-values, and prob> |t|are tabulated for the experimental and the fatigue model. It can be observed that the intercepts of the three samples are very close to each other, which proves the reliability of the fatigue test results. Moreover, prob> |t|values are very similar when comparing between the three samples. The degrees of freedom and goodness of fit for the fatigue samples and the fatigue model are shown in Table 7. It can be seen that the goodness of fit for the three samples is 98.3% on average, while for the fatigue model, it was 93.6%. These numbers show that the data fitting was conducted with a high level of accuracy. ANOVA was carried out for the fatigue samples and the presented model, as shown in Table 8. The sum of squares, mean squares, F-values, and Prob>F terms with p value less than 0.05 were considered as significant. The statistical parameters–sum of squares, mean squares, F-values, and Prob> F–were found to be close to each from for the fatigue samples, which agrees with the data presented in Table 6.

In previous related investigations, a more linear trend in the variation of fatigue life was observed in the semi-log plot for the other combinations of fibre-reinforced polymers such as basalt-reinforced epoxy, carbon-reinforced epoxy, Polyaniline nano fibres and flax-reinforced epoxy [67,68,69]. The observations in this research shows that the fatigue life of glass fibre-reinforced polyester composite materials significantly increase with a minimal reduction in the applied stress level, which means that the rate of increase in the fatigue life for glass fibre-reinforced polyester composite materials is more than that for glass fibre-reinforced epoxy composite materials and flax fibre-reinforced epoxy composite materials.

## 4. Conclusions

In the current investigation, the tensile behaviour and the tension–tension fatigue performance of glass fibre-reinforced polyester composite materials was studied. The influence of fibre volume fraction and the applied stress level on the fatigue life of composite materials were investigated through experimental and analytical modelling that lead to the following summarized points:

- The applied stress levels noticeably influenced the failure behaviour of glass fibre-reinforced polyester composite materials. The samples failed in stress concentration for an applied stress of 50% of the maximum tensile strength or below, while the failure was in pure tension because of the breakage of glass fibres for an applied stress of 65% of the maximum tensile strength or above.
- The 2D Halpin–Tsai model is the nearest prediction model to the experimental data for representing the Young’s modulus of the materials.
- The glass fibre-reinforced polyester composite materials exhibited 91% retention of its initial strength after 5 million fatigue cycles for the composition with the highest fibre volume fraction. The stiffness loss of the polyester composite samples can be reduced by avoiding distributing fibres in the transverse orientation.
- The fatigue-based analytical model presented in this research has taken into consideration the influence of fibre volume fraction, applied load, and the stress ratio, which helps to reliably predict the fatigue life of glass fibre-reinforced polyester composite materials at both very low cycles and very high cycles.

## Figures and Tables

**Table 1:** The mechanical and physical characteristics of unsaturated polyester.

Property	Value
Density	1.09 g/cm^3^
Modulus of Elasticity	3.3 GPa
Tensile Strength	40 MPa
Flexural Strength	45 MPa
Viscosity at 25 °C	250 cP

**Table 2:** The mechanical and physical characteristics of E-glass fibres.

Property	Value
Density	2.56 g/cm^3^
Modulus of Elasticity	72.5 GPa
Tensile Strength	2712 MPa
Refractive Index	1.55
Thermal Conductivity	1 W/m.K

**Table 3:** Predicted Young’s modulus values in MPa, for polyester and glass fibre-reinforced polyester composites.

Material	2D Halpin–Tsai	3D Halpin–Tsai	Rule of Mixtures	Inverse Rule of Mixtures
Neat Polyester (PE)	3300	3300	3300	3300
PE20GF	8675.24	6150.79	17,140	4078.59
PE35GF	12,477.85	8223.78	27,520	4955.47
PE50GF	16,099.86	10,243.34	37,900	6312.67

**Table 4:** Stress levels, stiffness loss, and failure mode of polyester composites.

Material	Stress Level	Stiffness Loss	Fatigue Life	Failure Mode
PE20GF	75%	2.44%	406	Pure tension
	65%	2.98%	1147	Pure tension
	50%	3.71%	2389	Stress concentration
	40%	4.88%	41,786	Stress concentration
	25%	3.27%	5,000,000	Test stopped at 5M cycles
PE35GF	75%	3.14%	406	Pure tension
	65%	3.78%	1147	Pure tension
	50%	4.86%	2389	Stress concentration
	40%	5.94%	41,786	Stress concentration
	25%	4.11%	5,000,000	Test stopped at 5M cycles
PE50GF	75%	3.67%	406	Pure tension
	65%	4.74%	1147	Pure tension
	50%	5.03%	2389	Stress concentration
	40%	5.99%	41,786	Stress concentration
	25%	4.29%	5,000,000	Test stopped at 5M cycles

**Table 5:** Schematic representation of the mechanical behaviour and attributed damage mechanisms in glass fibre-reinforced polyester composites based on varied fibre volume fractions and applied stress levels.

Material	Loading Condition	Failure Mode	Damage Mechanism
PE20GF, PE35GF, PE50GF	Static Tensile Testing	Pure tension	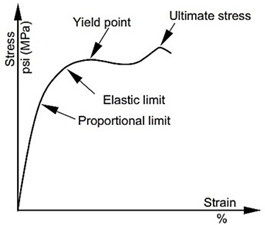
	Fatigue Testing at 75% of maximum tensile strength	Pure tension	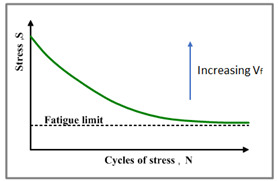
	Fatigue Testing at 65% of maximum tensile strength	Pure tension	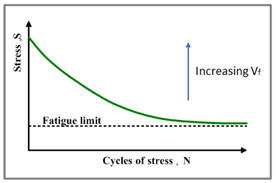
	Fatigue Testing at 50% of maximum tensile strength	Stress concentration	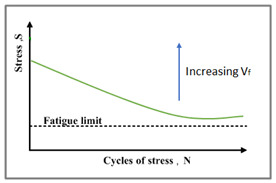
	Fatigue Testing at 40% of maximum tensile strength	Stress concentration	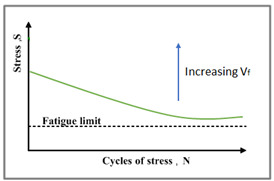
	Fatigue Testing at 25% of maximum tensile strength	Test stopped at 5M cycles	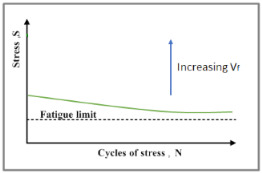

**Table 6:** Standard error and t-values for tension–tension fatigue samples.

		Value	Standard Error	*t*-Value	Prob>|t|
Sample A	Intercept	3.56 × 10^7^	8.02 × 10^2^	4.44054	0.14101
	B1	−2.50 × 10^7^	6.92 × 10^2^	−3.60993	0.17204
	B2	5.63 × 10^6^	1.84 × 10^2^	3.06436	0.20081
	B3	−4.09 × 10^5^	1.52 × 10^1^	−2.68689	0.22682
Sample B	Intercept	3.55 × 10^7^	7.90 × 10^2^	4.49047	0.13949
	B1	−2.48 × 10^7^	6.81 × 10^2^	−3.64579	0.17043
	B2	5.59 × 10^6^	1.81 × 10^2^	3.09178	0.19915
	B3	−4.067 × 10^5^	1.5 × 10^1^	−2.70892	0.22513
Sample C	Intercept	3.57 × 10^7^	8.10 × 10^2^	4.40927	0.14198
	B1	−2.51 × 10^7^	6.99 × 10^2^	−3.58738	0.17307
	B2	5.65 × 10^6^	1.86 × 10^2^	3.04713	0.20187
	B3	−4.11 × 10^5^	1.54 × 10^1^	−2.67311	0.2279
Model	Intercept	2.26 × 10^8^	3.89 × 10^2^	5.80045	0.0102
	B1	−1.67 × 10^8^	3.92 × 10^2^	−4.24969	0.02388
	B2	3.76 × 10^7^	1.10 × 10^2^	3.40608	0.04227
	B3	−2.64 × 10^6^	9.11 × 10^1^	−2.89251	0.06288

**Table 7:** Residual sum and R-square for tension–tension fatigue samples.

	Sample A	Sample B	Sample C	Model
Number of Points	5	5	5	7
Degrees of Freedom	1	1	1	3
Residual Sum of Residual	3.35 × 10^11^	3.25 × 10^11^	3.42 × 10^11^	5.39 × 10^14^
R-Square(COD)	0.98317	0.98366	0.98285	0.93624
Adj. R-Square	0.93268	0.93466	0.93141	0.87249

**Table 8:** ANOVA analysis of tension–tension fatigue samples.

		DF	Sum of Squares	Mean Square	F Value	Prob > F
Sample A	Model	3	1.96 × 10^13^	6.52 × 10^12^	19.47356	0.16471
	Error	1	3.35 × 10^11^	3.35 × 10^11^		
	Total	4	1.99 × 101^3^			
Sample B	Model	3	1.96 × 101^3^	6.52 × 10^12^	20.07146	0.16229
	Error	1	3.25 × 10^13^	3.25 × 10^11^		
	Total	4	1.99 × 101^3^			
Sample C	Model	3	1.96 × 101^3^	6.53 × 10^12^	19.10592	0.16625
	Error	1	3.42 × 10^13^	3.42 × 10^11^		
	Total	4	1.99 × 101^3^			
Model	Model	3	7.91 × 10^15^	2.64 × 10^15^	14.68494	0.0268
	Error	3	5.39 × 10^14^	1.80 × 10^14^		
	Total	6	8.45 × 10^15^			

## Data Availability

The data presented in this study are available in the article.
